# Not too sick, not too well: reducing the diagnostic void in pediatric emergency medicine

**DOI:** 10.1038/s41390-024-03598-2

**Published:** 2024-09-30

**Authors:** Damian Roland, Timothy Horeczko, Edward Snelson

**Affiliations:** 1 SAPPHIRE Group, Population Health Sciences, Leicester University, Leicester, UK; 2 Paediatric Emergency Medicine Leicester Academic (PEMLA) Group, Children’s Emergency Department, Leicester Royal Infirmary, Leicester, UK; 3 Los Angeles County-Harbor-UCLA Medical Center, Torrance, CA USA; 4 David Geffen School of Medicine at the University of California, Los Angeles, CA USA; 5 Children’s Emergency Department, Norfolk and Norwich University Hospitals, Norwich, UK

## Abstract

Emergency clinicians must rapidly evaluate the acutely ill or injured child. In a resource-stressed environment, “spotting the sick child” is essential for appropriate stabilization, treatment, and further management. Overlooking clinical features in a child’s presentation may impede timely care. Complicating factors include the volume of patients seeking care, unfettered access to emergency services, parental perceptions and expectations, and clinician biases. Notwithstanding, after an appropriate history and physical exam, some children do not fall under the standard rubric of “sick or not sick”. This article explores strategies to recognise the child who may lie in the diagnostic void between those who are obviously well and those who are not.

## Introduction

There is a considerable emphasis in pediatric emergency medicine on the task of recognising the unwell child. This focus tends to be based on decision making around individual patients and fails to acknowledge that emergency physicians are usually responsible for dealing with multiple patients at any one time. As such, the rapid recognition of the child with no emergent medical condition – the well child – is important in terms of mitigating iatrogenia, utilization of resources, and surge capacity for acutely ill and injured children. An additional benefit to the rapid discharge of children with non-emergent conditions is to the treating clinical team, in terms of offloading cognitive burden and workflow bottlenecks. We explore models for recognising wellness in order to improve the ability of clinicians to focus on the significantly unwell children in their care.

## Background

Over 30 million emergency department visits are made by children less than 18 years old in the United States yearly.^1^ Over 96% of children are treated and sent home. In pre-pandemic 2018, there were 100 visits per 100 persons aged 1 year and younger and 58 visits per 100 persons aged 1–4 years.^2^ During the same year, an estimated 34% of primary care visits made by children under 1 were for a “new problem”.^3^ Due to an array of systemic, demographic, and financial reasons the emergency department is increasingly becoming a destination of first choice for care, independent of acuity.^4,5^

Traditionally the emphasis in emergency medicine has been the recognition of the seriously unwell child with a secondary emphasis on recognising frank wellness. Attempts to give structure to the assessment of unwell children typically result in poor sensitivity and specificity. For example one UK primary care based study found that only 6% of children assessed using the traffic light system of signs and symptoms were categorised as “green”, that is, not requiring urgent intervention.^6^ This poor specificity likely results from the normal physiology of unwell younger children who have a tendency to extreme signs and symptoms during uncomplicated lower risk infections. The speedy recognition of children who are not requiring admission has clear benefits including improving the ability of emergency departments to reduce crowding and therefore focus more clinical time to those remaining. This in turn may improve the ability of teams to recognise and treat the seriously unwell children in their care.

## What is the impact of increasing use of the emergency department by children and young people?

Large patient volumes may saturate or overwhelm the capacity of clinicians, auxiliary staff, and space. The stressed environment may impact the triage and initial management of children presenting to the emergency department. A particular challenge is the distinction of an emergency medical condition (i.e. requiring immediate or urgent attention) from a medical complaint or concern that may be addressed outside of the emergency department. A good example of this is in self-resolving minor illness or injury. Most previously healthy children with viral syndrome do not require medical evaluation or professional treatment. When a family brings a child with a simple viral syndrome to the emergency department, there is an inherent discordance involving parental fear, parental convenience, disease severity, expectations of the visit, and the clinician’s assessment. Ever-increasing emergency department utilization amplifies the effect of every decision made by emergency clinicians, affecting patients, clinicians, and the system. It is imperative we reduce the decision-making load on acute and emergency clinicians.

The standard understanding is that the layperson determines the emergency. That may be true in terms of the decision to go to the emergency department but often has little to do with the presence of an emergency medical condition. Data both before^7^ and after^8^ COVID highlight parental perception of illness is often far higher than the child’s actual illness. However passing judgement on parental decision making risks paternalism, if it can be challenging for clinicians to recognise serious illness why not so parents? And this creates a dichotomy, on the one hand, asking the family to go the ED purely for emergencies may risk the ill child staying at home and worsening. On the other hand, the current use of the ED as an entry point to the medical system is not sustainable and may put emergent patients at risk.

Take for example young Anya (case study: Part A)

*Anya is 18 months old. She has had a cold for a couple of days. This morning her mother finds her to be pale, clingy, and not her usual self. She vomited once, and now refuses food. Anya is brought to the emergency department*.

*She is seen on arrival and noted to be flushed and quiet. She is aware of her surroundings and reaches for objects. She has a raised respiratory rate (42 breaths per minute) and a high heart rate (165 beats per minute). There is no rash but her hands and feet feel cold. Her temperature is 40.1 degrees centigrade. She had spat out the acetaminophen (paracetamol) her mother had tried to give her in the morning*.

What now? Does Anya need to be prioritized, or is she safe to wait? Is Anya going to require investigations or admission?

Given that febrile illness is the second most common presentation to Emergency Departments (after breathing difficulty) it is easy to see why this type of case poses a challenge. Fever in the young child can markedly affect their appearance, a cardinal feature in the initial assessment of children. Compounding the initial assessment are the parent or caregiver’s preconceived notions of the significance of fever.^9,10^ On the one hand, clinicians aim to ally with parents to take their concerns seriously. On the other hand, the dissonance between perception of illness and safety risk of fever in children between parents and clinicians is often marked.^11,12^

In the presented scenario, historically, protocols would flag this child is at risk of sepsis.^13^ The clinician may elect to observe or even undertake initial investigations. If parental concern was strong and the treating clinician felt that Anya’s overall appearance was not in keeping with a virus they may also elect to treat with antibiotics awaiting the results of various cultures. Regardless of the path chosen, the clinician wrestles with a feared retrospective criticism if the child were to return more unwell with a confirmed bacterial illness.

## Sepsis: through a glass darkly

Sepsis is a dynamic disease process. Clinicians see the individual patient at various stages of illness. A healthy child’s physiologic response to both benign viral syndrome and early bacteremia may be identical, especially early in the course. Viral infections often cause prime conditions for a subsequent bacterial superinfection, also with varying degrees of acuity. Progression to sepsis is rare and unpredictable; screening every child with invasive testing is not warranted.^13,14^

Uncertainty, patient volumes, medicolegal risk, established practice patterns, and increasing reliance of the healthcare system on emergency medical services run in contradistinction to the above ideal practice pattern. The highly sensitive and non-specific systemic inflammatory response syndrome (SIRS) schema has been used with mixed results.^15^ SIRS became protocolized in many institutions, resulting in “SIRS alerts” and mandatory, albeit low-yield testing.^16^ Even in the high-risk population of children under three months of age, the presence of SIRS was not predictive of invasive bacterial illness.^17^

The Phoenix pediatric sepsis criteria were developed as a response to over-testing and over-utilization of inpatient resources.^18^ The paradigm has shifted from physiological response to assessment of end-organ risk (Table 1). These definitions are far more specific than the previous screening criteria that were associated with a systemic inflammatory response syndrome (SIRS); the previous method of determining which patients needed intervention.

**Table 1:** Contributions to the Phoenix sepsis score

Variables	Ranges of points score	Physiological value used
Respiratory	0–3	Pa02 or SpO2:FiO2
Cardiovascular	0–6	Number of vasoactive Medications Lactate Mean arterial pressure by age
Coagulation	0–2	Platelets International normalised ratio D-Dimer Fibrinogen
Neurologic	0–2	Glasgow coma score

The vast majority of children who present to Emergency Departments will not have sepsis.^19,20^ Even at current vaccination rates, children over 3 months of age (presenting to a Children’s Emergency Department) display a rate of serious bacterial infection of less than 7%.^21^ The majority of bacterial infections follow an uncomplicated course with sepsis being a rare outcome. Finding the ‘sepsis’ needle in the ’emergency department’ haystack is a daily task. This is particularly true for the children aged between 6 months and 6 years old due to their tendency to have more exaggerated changes in physiological parameters when unwell with an uncomplicated infection. A previously healthy 2-year-old who initially appears miserable with volume depletion and tachycardia has reasonable potential to improve rapidly with hydration. Serial examinations during an observation period are important to track his recovery or to make the decision to intervene further. The need for sequential review in an overcrowded department creates a high-risk, decision-rich environment which is a significant cognitive and emotional load.

## Do we need a change of focus in spotting the unwell child?

There is no standardized definition of wellness available to health care professionals. To recognise the well child requires training, time, and thought. Parental descriptions and use of terminology concerning the child’s condition may not match that of the clinician.^8^ Age, duration of symptoms, and access to care outside of the emergency department all influence how quick an emergency physician may be to deem a child “well” (read: safe for discharge home). From a systems lens, emergency departments could not function if every child with normal vital signs and common, benign, self-limiting condition received an in-depth invasive investigation. From an interpersonal lens, there may be a dissonance between the wellness that the emergency physician sees and the miserable symptomatology the parent experiences.^22^ Common scenarios include presentations for fever, cough, rhinorrhea, vomiting, or refusal to eat.

The effect of fever in particular on decision making is complex. Fever is a common feature of uncomplicated low risk illness and is non-specific. Due to a weak association between higher fever and serious bacterial infection many guidelines include a fever threshold as a discriminating feature in decision making. Unfortunately fever is too non-specific to have any clinical usefulness, regardless of its degree. In practice fever does influence the decision-making process due to the features commonly associated with a raised temperature: typically tachycardia and altered peripheral perfusion, alongside a reduced activity level and changes in behaviour. While fever itself is non-specific the overall appearance and behaviour of a child that is febrile at the time of assessment is often one that impairs the decision making of the clinician.^23^ For this reason well timed analgesia can be an extremely effective strategy in the emergency department, allowing a clinician to see a child who had a high fever an hour ago with all the associated features of sepsis yet now looks and behaves like a well child.

The Phoenix score, with its focus on evidence of organ dysfunction is an important progression in our approach to recognising true sepsis. However to achieve this the Phoenix score requires extensive blood work. In pediatrics the ideal is to avoid the trauma of venepunture wherever possible. We therefore need a way of deciding when organ dysfunction is very unlikely. One model that works well for recognition of the well child is to focus on activity and behaviour.^24^ In paediatric practice these features give valuable clinical information about the current metabolic status and end organ function of the child being assessed. While non-specific features such as cough and fever support a process of differential diagnosis (upper or lower respiratory tract infection) the behaviour and actions of the child give important information about the clinical effects of the infection regardless of what the diagnosis is. A child with fever and a cough who is seen to be running around, eating, drinking, playing and chatting is demonstrating metabolic and cardiorespiratory sufficiency. Arguably this supersedes the distinction between upper and lower respiratory infection as a lower respiratory tract infection with minimal clinical effect can be managed conservatively in a similar way to upper respiratory tract infection.^25^

This model of predominantly using activity and behaviour for recognition of wellness essentially relies on the sensitive nature of higher cerebral function. The brain has a high metabolic requirement and function is much more quickly affected by any lack in perfusion, hypoxia or hypoglycemia than other vital organs. This is particularly true for higher cerebral function which is affected first while hindbrain function is preserved for longer when the brain is under stress. This physiological model explains why it is valid in clinical practice to allow an interactive smile^24^ to be an extremely valid examination finding.

There are two relevant factors to an approach that focuses on spotting wellness. The first is that some ‘well appearing’ children need to be in an emergency department (think safeguarding or oncology) and the second is that rapid recognition of the well child may be challenging in children who are neurologically atypical at baseline.^26,27^ It has been increasingly recognised that children with neurodivergence or neurodisability are at significant risk for poor outcomes when they become ill.^28^ Clinicians may misjudge wellness for an abnormality, and vice versa. For health care professionals assessing children who are not neurotypical, it is important to be aware of the specific difficulty and risk in the emergency department setting. A parent or carer who knows the child well and is able to verbally compare current state to the child’s baseline activity and behaviour can be an important substitute for a direct comparison of observation to a presumed normal. This requires trust by the health care professional of the caregiver’s judgement and experience.

## Decision making in the diagnostic void

Decision making can be straightforward at the extremes of the well to unwell spectrum. Non-specific signs or symptoms may sway the clinician to investigate further. If after treatment and reassessment the child “proves” his wellness (i.e., normalized vital signs, improved appearance and activity), then intervention can be de-escalated. Conversely, concerning signs such as severe tachycardia, cold peripheries, poor responsiveness, and inactivity flag the child as unwell; he is at high-risk for decompensation even after initial partial improvement with resuscitative measures. What commonly creates a dilemma in paediatric emergency medicine is the child with a mixed picture, the “in-betweener”. Typically this is a child who is alert and active but tachycardic and symptomatic.

As there is no single and universally accepted strategy for decision making in such children a variety of approaches exist. These include:

- Treat and re-evaluate
- Risk stratify with biomarkers
- Treat and admit

## Treat and re-evaluate

This strategy is commonly used in emergency departments. The validity of this approach stems from the dilemma that, especially in young children, physiological response to uncomplicated illness is often extreme and indistinguishable from physiological compensation or decompensation due to sepsis.^29^ Given no high-risk medical comorbidities, the previously well child with a self-limiting viral illness may respond well to this strategy.

In this approach, administration of an antipyretic or analgesic is being used more as an investigation based on the presumption that the physiological changes due to the illness will return to baseline while dysregulated parameters and signs of organ dysfunction will remain deranged. This approach works well when any abnormal parameters make a significant positive change and this correlates with a healthy activity level. The pitfalls of this approach are many including a lack of clear parameters for success. Does the heart rate need to return to normal or is a degree of normalisation sufficient? This is particularly problematic when the reassessment is handed over to a different person (for example following a change of shift) as this model of analgesia and review works best when the same person sees the effect of analgesia directly.

## Risk stratify with biomarkers

The use of biomarkers (e.g. CRP or procalcitonin) in decision-making is often used in patients with some risk of decompensation or invasive illness, such as young infants.^30^ Their use in older, previously well children is debated.^31^ Given the range of presentations and medical complexity of patients who present to the ED, it is tempting to shed cognitive load and depend on an objective test. Applicability of the test depends on its performance, or accuracy. Accuracy is a function of prevalence of the disease as well as the sensitivity and specificity of the test itself. While biomarkers do have some correlation with the significance of infection there are no clear thresholds which allow for these tests to reliably rule out or rule in serious bacterial infection. Biomarkers also give no information about the clinical effect of an illness. If there were any test or formula that had good sensitivity and specificity it would be the gold standard approach. Since no such test exists biomarkers tend to be used in those cases who are neither well enough to immediately decide to discharge nor unwell enough for an immediate decision to treat. The very decision to use a biomarker is a critical intervention in itself and dependant on the experience of the clinician, the clinical context of the decision and the prevalence of disease.^32^

We acknowledge the role of biomarkers in validated algorithms, where the biomarkers allow determination of an action as a critical decision making node when a disease process has been identified. However, using biomarkers in a defensive fashion for a well, vaccinated child with likely viral source only causes harm to the patient, increases costs, and delays the care of other, sicker children.^33^ Therefore while biomarkers appear to have inherent face validity they are not always necessary.

While little work has been done that could claim to compare the effectiveness of the different approaches there is interesting proxy evidence that suggests clinical decision making alone is not just valid but as safe and more efficient than adding biomarkers to the decision making process. The Petechiae in Children (PIC) study^34^ used local guidelines for children with fever and petechial rash and applied these guidelines to a large dataset of children with fever and non-blanching rash. The original paper demonstrated that all of the local guidelines used had better specificity than the current national guideline (which essentially used a blanket treat and decide approach) without losing sensitivity. All of the guidelines using biomarkers in decision making achieved a specificity of up to 36%. Examination of a different guideline which relied wholly on clinical decision making without the use of biomarkers improved specificity to 69% without the loss of any sensitivity.^35^ This highlights a need to do similar research comparing different approaches to decision making in all febrile children and not just those with non-blanching rash. Ultimately given the PIC study demonstrated petechiae are no longer a risk factor for sepsis in a population vaccinated against meningococcus, it is arguable that this publication has shown that clinical decision making without the use of biomarkers is both safe and perhaps more effective in some populations.

## Treat and admit

This approach involves an early decision to admit the child with treatment ongoing. The benefit to the child would be to allow further treatment and testing and to assure a safe disposition from the emergency department. In an overburdened system, routing obviously ill children to other available clinicians may be a shrewd strategy. Early anchoring^36^ of the not-so-sick child (or one who could benefit from the above treat and reassess or risk-stratify strategies) may conversely lead to overtreatment^37^ and could overwhelm inpatient capacity.

A default treat-and-admit approach is most valid in high-risk groups where the specificity will be greatest. For example, a well appearing, febrile, and tachycardic 3 day old is very high-risk for serious bacterial infection whereas a well appearing, febrile, and tachycardic 3-year-old is very low-risk. This is because there is an incidence of invasive bacterial illness in a well appearing 3 day old just on the basis of having a fever, whereas the well appearing 3 year old with a fever has a negligible (but not non-existent) risk of invasive bacterial illness. Both cases demonstrate an innate vigorous physiological reaction to fever. The 3-day-old, however, is at high-risk for decompensation and serious sequelae; the three-year-old has proven his robustness to recover. If high fever and vital sign abnormality were the deciding (read: anchoring) factor to admit regardless of emergency department course, harm may be done to those who would not benefit from admission.

## Reducing the void using an early warning system decision tree

Traditionally the approach has been to sift out the sickest children through senior staff, triage and/or Early Warning Systems and then work through the remaining equivocal cases.^38^ Early Warning Systems have become synonymous with Early Warning Scores i.e. individual numerical scores based on physiology which correspond to various levels of escalation however Early Warning Systems are much broader strategies to recognise unwell children. They incorporate not only physiological measurements through the use of scores but require the use of subjective judgements of staff, and increasingly incorporate the views of the caregivers.^39^ Furthermore they are predicated on healthcare cultures which are not beholden to hierarchy and utilise communication processes aligned with human factors theory. A combination of effective decision-making approaches which increase the number of well children discharged early in the patient journey, allow prompt treatment of the most unwell and maximise focus on the group in the middle (the void) is needed. The delivery of this is complex given it requires multiple staff members and a patient group who may have evolving disease process.

**Fig. 1:**
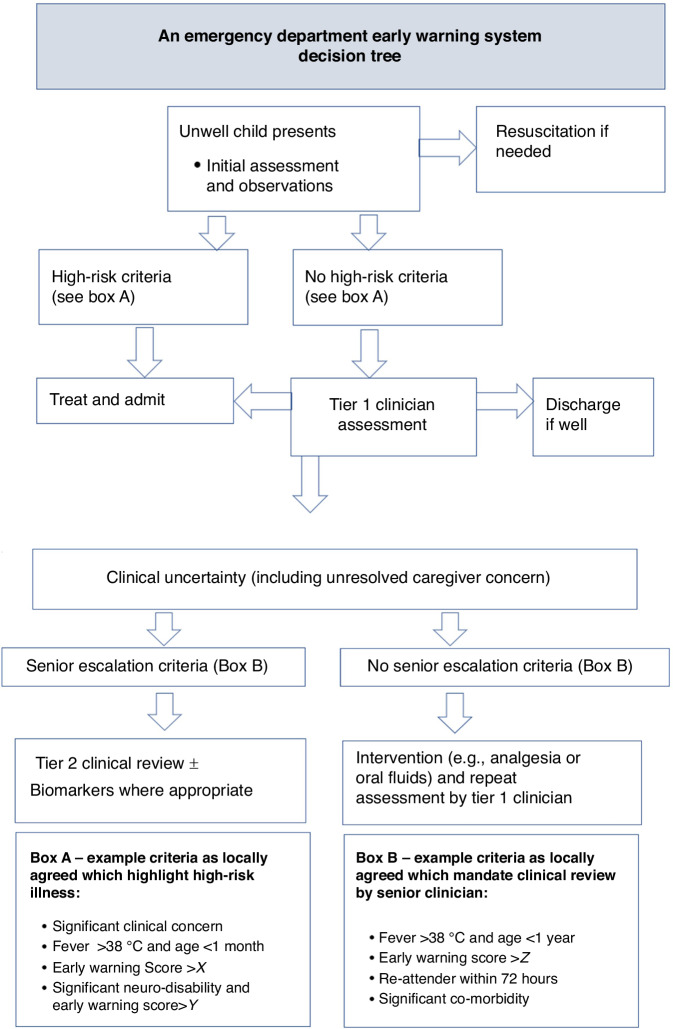
An early warning system decision tree approach to decision making in the Emergency Department.

Figure 1 brings together an early warning system decision tree approach to decision making in the Emergency Department. At its heart is the use of clinical expertise to determine the disposition of patients, but in a tiered approach, so that not all decisions need to be made by the most senior personal. Early Warning Scores, biomarkers and caregiver concern all have a role to play but are adjuncts within a decision tree which aims to remove the most well and unwell from the patient load and thereby reducing the cognitive strain by working in the void area.

The application of the decision tree may result in the following conclusion to the case study

Case Study Part B (Evaluating Risk)

*Anya has no high risk criteria (Box A). The initial clinical evaluation, performed by a junior doctor finds that Anya has an upper respiratory tract infection. Due to the abnormal physiological parameters (Box B) the junior consults a senior doctor who reviews Anya. The senior’s opinion is that Anya’s peripheral coldness, raised heart rate and respiratory rate are explained by a combination of fever and discomfort. They recommend a period of observation to re-evaluate after the analgesia has had an effect*.

## A culture of leaving well enough alone

A fundamental aim of pediatric care is to seek out sick children and prioritize them. This culture ensures the delivery of education, and of support, departments must audit and evaluate near-miss cases. This leads to a predisposition that children are presumed ill and that perhaps an overemphasis on that fact that every discharge home carries some degree of risk. Because acute care presentations often occur at the beginning of a disease process, clinicians vary in their comfort with uncertainty or risk tolerance.^40^ This experience is often difficult to teach and so communities of practice and other ongoing professional development strategies may best help clinicians understand the practice patterns of their peers. Opportunities to discuss, adjust or update practices are multiple, regardless of locale.^41^

The careful clinician can only make the best decision with the information and resources at the time of presentation. The risk of decompensation of a discharged patient may only removed by admitting all children, negating the value of a primary assessment service. For the risk to be acceptable we must avoid criticism of the decision to discharge where a valid clinical assessment and decision process has taken place. Instead it is key to accept that rarely a child who appears well will later become unwell. “Sick Children look Sick” wrote Green et al.^14^ nearly a decade ago. While the medico-legal implications of discharging a child for them to return more unwell are significant this occurrence does not mean the initial discharge decision was incorrect. In order to protect patients and staff the discharging clinician must provide excellent verbal and written safety netting advice.^42,43^ By doing so we employ the parent or carer in an ongoing decision-making process that is as dynamic as the illnesses with which children present. The well-child visit to the emergency department may also be a teachable moment^44^ to engage parents in what clinical setting best matches common complaints (e.g., clinic, urgent care, or emergency department).

Case Study part C (Resolution)

*Anya received acetaminophen per rectum and the emergency physician confirmed with her parents that she had no high-risk features. Shared decision-making included a short observation time and reassessment. Forty-five minutes later, Anya had defervesced; she was taking fluids and hungrily eating a cookie. Her repeat vital signs showed a heart rate of 135 beats per minute and a respiratory rate of 32 breaths per minute. The emergency physician was transparent in his logic to the parents and cautioned that although there is still a risk of bacterial infection now or in the near future, home monitoring was a safe first step. Careful precautionary advice was given. The family was discharged home, and the emergency physician was immediately called to the bedside of an apneic infant*.

## Conclusion

The emergency care system is overloaded and above capacity. The mission of emergency departments to care for acutely ill and injured children is in conflict with the present-day clearinghouse phenomenon in which the spectrum of presentations is widely expanded to include many well children. In terms of preservation of mission, resources, and reducing harm, spotting the well child will become increasingly valuable. Clinician awareness of societal needs, patients’ access to healthcare, and a culture of satisfaction are substantial barriers to re-routing patients to the best venue for medical attention. A cultural shift is needed to enable prompt decision making regarding children at lowest risk of serious illness. In addition to institutional support, this will depend on adequately experienced clinicians to make decisions based on clinical judgement rather than biomarker output.
